# Interacting and joint effects of triglyceride-glucose index and blood pressure on cardiovascular diseases risk: a prospective cohort study

**DOI:** 10.1186/s13098-024-01433-6

**Published:** 2024-08-02

**Authors:** Haozhe Cui, Qian Liu, Zhiming Zhao, Xiangming Ma

**Affiliations:** 1 School of Medicine, Nankai University, Tianjin, China; 2 Department of Cardiology, Kailuan General Hospital, Tangshan, China; 3 Department of Hepatobiliary Surgery, Kailuan General Hospital, Tangshan, 063000 China; 4 The Faculty of Hepatopancreatobiliary Surgery, The First Medical Center, Chinese People’s Liberation Army General Hospital, Beijing, China

## Abstract

**Aims:**

The triglyceride-glucose (TyG) index and hypertension (HTN) are established risk factors of CVD. However, there is a dearth of studies investigating the synergistic influence of the elevated TyG index and HTN on CVD risk, as well as any potential interaction between these factors.

**Method:**

For this investigation, we enlisted 88,384 individuals from the Kailuan Study who did not have a history of stroke, myocardial infarction, or cancer at baseline. Incidences of CVD between 2006 and 2021 were confirmed through a thorough review of medical records. Participants were categorized into 6 groups according to BP status(normal/elevated BP, stage 1 and stage 2) or the TyG index(low and elevated group), respectively. The Cox proportional hazard regression models were used to assess the association of BP status and TyG index with incident CVD. The multiplicative and additive interactions were also determined.

**Results:**

Following a mean follow-up period of 13.66 ± 3.24 years, incidents of CVD, MI, and stroke were observed in 8,205, 1,728, and 6,705 individuals, respectively. The BP category and TyG index additively increased the risk of CVD, MI and stroke. There were significant interacting and joint effects of TyG index and BP status on CVD risk. Additionally, stratification analysis further confirmed that the relative contribution of hypertension to the CVD development decreased with deteriorating TyG index and that of TyG index was attenuated with increasing BP status.

**Conclusion:**

Our study demonstrated that a significant interaction between TyG index and BP status on the risk of CVD.

**Supplementary Information:**

The online version contains supplementary material available at 10.1186/s13098-024-01433-6.

## Introduction

Cardiovascular diseases (CVD) are prevalent non-communicable diseases, contributing to approximately one-third of all deaths worldwide [1]. The incidence of CVD has increased significantly over time, with cases nearly doubling from 271 million in 1990 to 523 million in 2019. Furthermore, CVD-related deaths have soared from 12.1 million in 1990 to 18.6 million in 2019 [2]. This alarming trend may be attributed to various modifiable risk factors, such as elevated body mass index, elevated systolic blood pressure, smoking, etc. [3]. Several countries, including China, are experiencing an enormous burden of CVD due to the escalating prevalence and inadequate management of cardiovascular risk factors [4]. Despite China having made tremendous progress in lifestyle modifications and treatment recently, substantial disparities persist between current trend of high prevalence of CVDand the recommended goals outlined in the guidelines [5]. Accurate assessment of patient’s long-term risk for CVD requires a more comprehensive approach than relying solely on a single indicator. Since CVD often develop due to the combined influence of multiple co-existing risk factors, a single measurement may not provide a sufficiently precise picture.

Insulin resistance (IR) and hypertension (HTN) are established risk factors of CVD that participate in the process of disease initiation and progression. Prior studies have identified relationships between IR and HTN, indicating that the combination of both factors may improve the stratification of CVD risk [6, 7]. The triglyceride-glucose (TyG) index, calculated from fasting blood glucose (FBG) and triglyceride (TG) levels, has demonstrated a strong association with IR and is considered a reliable and easily interpretable surrogate marker for IR [8]. Furthermore, multiple cohort studies have indicated that the TyG index may serve as a risk factor for the onset of CVD [9, 10]. The European Society of Cardiology (ESC) has updated 2023 guidelines for the management of blood pressure (BP) in patients with diabetes [11]. However, the potential impact of HTN on CVD risk remains uncertain in individuals with IR who do not progress to hyperglycemia or diabetes. Besides, whether the degree of IR is associated with increased risks of incident CVD in HTN patients remains to be fully understood [12]. Currently, there is a dearth of studies investigating the synergistic influence of high IR and HTN on CVD risk, as well as any potential interaction between these factors. The present study utilized data from a substantial community-based prospective cohort in China to evaluate the influence of BP status (normal/elevated, stage 1 HTN, and stage 2 HTN) and TyG index (low group and elevated group) on the likelihood of developing CVD, specifically myocardial infarction (MI) and stroke. The potential interaction between BP status and TyG index and CVD risk was also investigated.

## Methods

### Study participants

The present study utilized data from the Kailuan Study, a comprehensive community-based prospective cohort study. A comprehensive description of the study design and methodology is provided in a previous study [10]. A total of 101,510 participants (81,110 males and 20,400 females) aged between 18 and 98 years were recruited. These individuals underwent a series of assessments, including physical examinations, clinical and laboratory evaluations, and questionnaire interviews encompassing variables such as income, educational attainment, alcohol consumption, etc. Subsequently, anthropometric, cardiovascular risk factor, and self-reported questionnaires were conducted biennially from January 2006 to December 2021 (2006/07, 2008/09, 2010/11, 2012/13, 2014/15, 2016/17, 2018/19, and 2020/2021). Participants with heart attack, stroke, cancer, missing data on certain health indicators, or white coat HTN were excluded. A total of 88,384 participants were included in the final analysis (Figure S1).

The study adhered to the principles outlined in the Declaration of Helsinki and was approved by the Ethics Committee of Kailuan General Hospital. All participants willingly consented to participate in the study and provided written informed consent.

### Data collection and definitions

Data on demographic information, lifestyle behaviors, and clinical characteristics were obtained through a self-administered questionnaire, as described [13]. Education levels were stratified into primary or lower, middle school, and high school or higher. Smoking status was dichotomized as a current smoker or never/former smoker. Physical exercise was defined as participating in physical activity at least four times per week, with each session lasting a minimum of 20 min. Body mass index (BMI) was computed as weight in kilograms divided by height in meters squared (kg/m^2^). Diabetes was defined as an FBG level of ≥ 7.0 mmol/L, self-reported diabetes history, or use of antidiabetic medication.

Blood samples were collected in EDTA tubes in the morning following a minimum 8-hour fasting period. Biochemical measurements, including TG, high-density lipoprotein cholesterol (HDL-C), low-density lipoprotein cholesterol (LDL-C), high-sensitive C-reactive protein (Hs-CRP), FBG, and uric acid (UA), were analyzed using the Hitachi 747 autoanalyzer (Hitachi, Tokyo, Japan).

### BP category

BP was assessed in the left upper arm utilizing a calibrated mercury sphygmomanometer while the participant was in a seated position. Following a 5-minute rest period, a minimum of two BP readings were obtained. If the variance between the two measurements was ≥ 5 mm Hg, an additional measurement was taken, and the average value was calculated.

HTN status was determined according to the 2017 American College of Cardiology (ACC)/American Heart Association (AHA) guideline. Participants were classified into three groups based on their BP status: normal/elevated BP, stage 1 HTN, and stage 2 HTN. Normal/elevated BP was defined as systolic blood pressure (SBP) < 130 mmHg, diastolic blood pressure (DBP) < 80 mmHg, or not currently under antihypertensive medication; stage 1 HTN was defined as SBP of 130–139 mmHg or DBP of 80–89 mmHg; and stage 2 HTN was defined as SBP ≥ 140 mmHg or DBP ≥ 90 mmHg or the use of antihypertensive medication [14].

### Evaluation of the TyG index

The TyG index was calculated as ln (fasting TG [mg/dL] × FBG [mg/dL]/2), as previously described [10]. Participants were divided into two groups based on the median of the TyG index (8.57): the elevated group (TyG index > 8.57) and the low group (TyG index ≤ 8.57).

### Assessment of CVD

The study concluded after the occurrence of the first instance of CVD, all-cause mortality, or after the follow-up on December 31, 2021, whichever event transpired first. CVD, encompassing MI and stroke, were identified using the International Classification of Diseases 10th Revision (ICD-10) codes. The database of CVD diagnoses was cross-referenced with the Municipal Social Insurance Institution and Hospital Discharge Register, with annual updates conducted throughout the follow-up period. An expert panel was convened to meticulously review and validate annual medical records from local hospitals to differentiate patients with confirmed CVD from those with suspected CVD.

### Statistical analysis

The analysis of variance (ANOVA) and Kruskal-Wallis tests were employed to compare continuous variables, whereas chi-square tests were utilized for comparing categorical variables. The cumulative incidence of CVD was calculated using the Kaplan-Meier method.

Firstly, participants were stratified based on BP status (normal/elevated BP, stage 1 HTN, and stage 2 HTN) and TyG index (low group and elevated group) to investigate the relationship between TyG index and BP status and CVD risk. In the analysis of the combined effect, all subjects were assigned into six groups. Normal/elevated BP and low TyG index groups were used as a control group. Hazard ratio (HR) and 95% confidence interval (CI) for incident CVD were calculated, and adjustments were made for the same covariates used in the multivariable Cox regression analyses. Exposure factors were introduced into the multivariate model as an interaction term to test the interaction between HTN and elevated TyG index. An interaction term, created by multiplying the TyG index and BP levels (normal/elevated BP, stage 1 HTN, stage 2 HTN), was added to the model to assess multiplicative statistical interactions. Relative excess risk due to interaction (RERI), proportion of disease attributable to interaction (AP), and synergy index for interaction (SI) were calculated following the established methods [15, 16]. The significance of interaction was determined based on the non-inclusion of 0 and 1 within the 95% CIs for RERI, AP, and SI, respectively.

Subsequently, the relationship between BP status and incident CVD was examined based on TyG index grouping, dividing the study population into low and elevated groups. Cox regression analyses were performed to ascertain the correlation between BP categories and the occurrence of CVD, with adjustments made for baseline confounders such as age, sex, income, physical exercise (active or inactive), educational level, drinking (current or no), smoking (current or no), diabetes, anti-hypertensive medication, anti-diabetic medication, anti-hyperlipidemic medication, BMI, resting heart rate (RHR), LDL-C, UA, and Hs-CRP. Additionally, a comparative analysis of the relationship between the TyG index and incident CVD was conducted based on BP status. The study population was stratified into three groups: normal/elevated BP, stage 1 HTN, and stage 2 HTN, and adjustments were made for consistent confounders. Relative risk reduction (RRR) was estimated along with its corresponding 95% CI, and adjustments were made for covariates included in the multivariable Cox regression model. The RRR was calculated using the formula: (HR − 1)/HR.

Meanwhile, multiple sensitivity analyses were conducted to ensure the reliability of our findings. Events within the initial 2-year follow-up period were excluded to mitigate the risk of reverse causation. Subsequently, individuals with diabetes or those undergoing treatment with lipid/glucose-lowering medication were excluded from the main analysis. Additionally, the interactive and combined effects of diabetes status and BP status on CVD risk were evaluated.

All analyses were performed using SAS 9.4 (SAS Institute, Cary, NC), at a two-tailed alpha level of 0.05.

## Results

### Baseline characteristics

A total of 88,384 eligible participants were enrolled, with a mean age of 51.07 ± 12.39 years. Approximately 79.36% (70,142/88,384) were males. The average TyG index was 8.65 ± 0.69. The baseline characteristics of participants based on their TyG index and BP status are displayed in Table 1. The baseline characteristics of participants categorized by the TyG index are presented in Table S1. The baseline characteristics of participants categorized by BP status are presented in Table S2. Compared with individuals in the low TyG index and normal/elevated BP groups, those in other groups exhibited a higher likelihood of being older, male, less educated, current smokers and drinkers, having a higher prevalence of HTN and diabetes, taking anti-hypertensive medication, anti-diabetic medication, anti-hyperlipidemic medication, and possessing a higher BMI, LDL-C, RHR, UA.

**Table 1:** Baseline characteristics of participants by TyG index and BP status

	Normal TyG index			Elevated TyG index			*P*
	Normal	Stage 1	Stage 2	Normal	Stage 1	Stage 2	
N	15,420	14,745	14,128	8576	13,804	21,711	<0.01
Age, years	45.59 ± 13.01	49.37 ± 12.17	56.55 ± 11.49	48.04 ± 12.11	49.46 ± 11.25	54.76 ± 10.89	<0.01
Male, %	64.75	78.20	86.15	76.24	83.27	84.87	<0.01
SBP, mmHg	109.53 ± 10.10	122.76 ± 8.18	148.39 ± 17.22	111.77 ± 9.77	123.56 ± 7.98	148.28 ± 17.89	<0.01
BMI, kg/m2	23.04 ± 3.10	23.98 ± 3.18	25.03 ± 3.37	24.94 ± 3.15	25.64 ± 3.23	26.68 ± 3.41	<0.01
RHR, bpm/min	71.56 ± 9.521	72.92 ± 9.53	74.23 ± 10.23	72.95 ± 9.86	74.08 ± 9.70	75.98 ± 10.77	<0.01
LDL-C, mmol	2.17(1.70–2.67)	2.29(1.76–2.79)	2.40(1.85–2.90)	2.34(1.89–2.80)	2.39(1.90–2.85)	2.43(1.94–2.93)	<0.01
UA, U/L	269.30 ± 74.16	271.16 ± 72.58	286.08 ± 79.04	298.89 ± 83.32	297.87 ± 83.71	307.93 ± 91.13	<0.01
Hs-CRP, mg/dl	0.60(0.21–1.60)	0.60(0.20–1.60)	0.85(0.31–2.30)	0.80(0.32–2.03)	0.80(0.30-2.00)	1.06(0.41–2.60)	<0.01
TG, mmol	0.85 ± 0.27	0.90 ± 0.27	0.95 ± 0.26	2.25 ± 1.38	2.45 ± 1.63	2.50 ± 1.63	<0.01
FBG, mmol	4.90 ± 0.68	4.96 ± 0.71	5.00 ± 0.79	5.78 ± 2.07	5.83 ± 1.96	6.16 ± 2.17	<0.01
TyG index	8.04 ± 0.35	8.12 ± 0.33	8.18 ± 0.31	9.09 ± 0.47	9.17 ± 0.51	9.23 ± 0.54	<0.01
Smoking, %	29.83	29.50	28.77	36.63	32.89	30.06	<0.01
Drinking, %	13.61	16.35	20.04	17.04	19.32	19.97	<0.01
Diabetes, %	1.23	1.36	2.69	11.60	12.61	19.93	<0.01
Active exercise, %	13.21	13.41	19.27	13.70	12.58	17.26	<0.01
Anti-hypertensive medication, %	0	0	23.16	0	0	27.68	<0.01
Anti-hyperlipidemic medication, %	0.27	0.20	0.92	0.82	0.32	1.71	<0.01
Anti-diabetic medication, %	0.56	0.38	1.24	3.18	2.35	4.77	<0.01
High school or above, %	34.29	18.72	12.75	30.07	18.97	14.14	<0.01

SBP systolic blood pressure, BMI body mass index, RHR resting heart rate, LDL low-density lipoprotein, UA uric acid, Hs-CRP high-sensitivity C-reactive protein, TG triglyceride, FBG fasting blood glucose, TyG triglyceride glucose

### Combined effects of TyG index and BP status on CVD

**Fig. 1:**
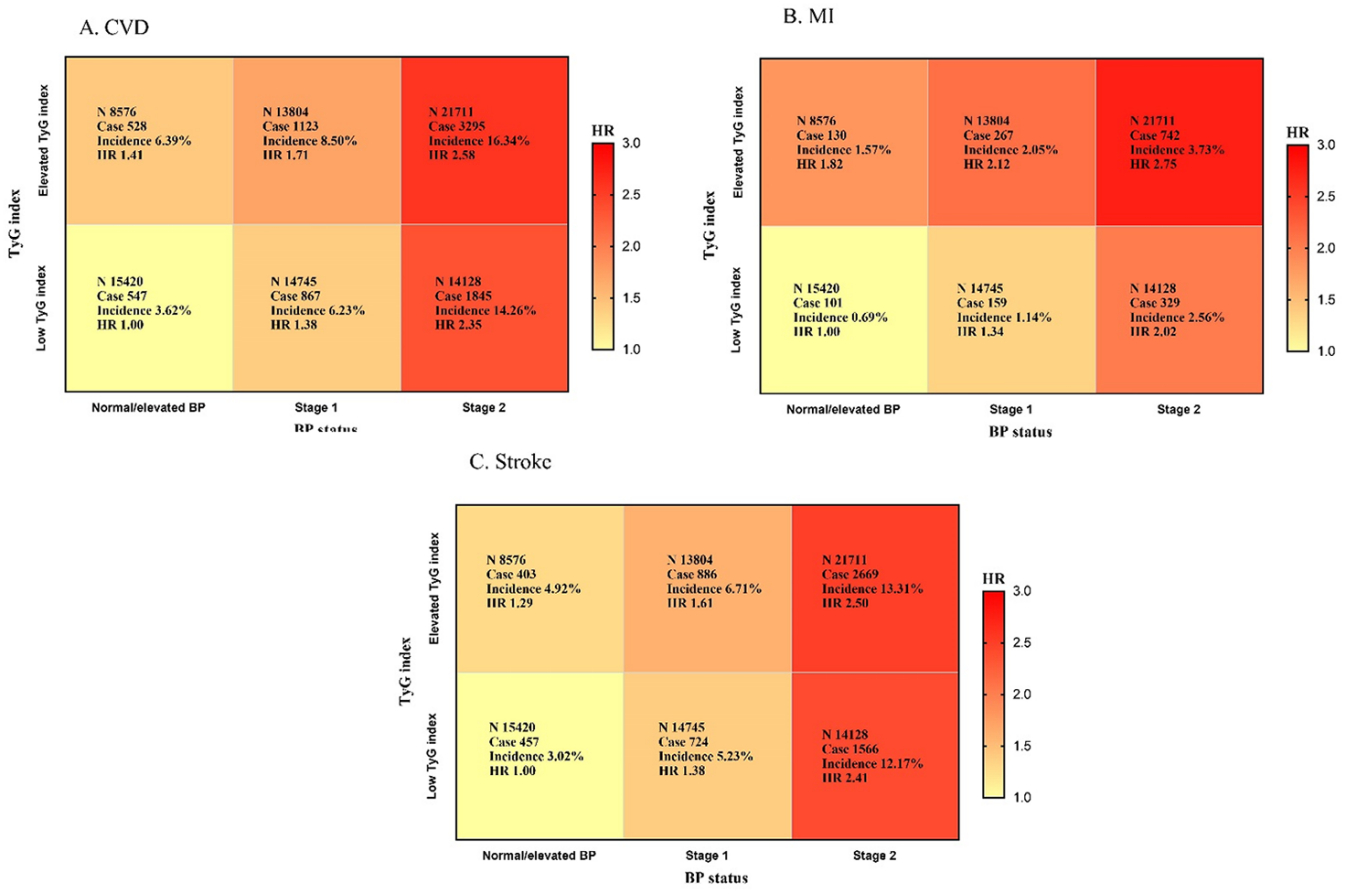
Combination of TyG index and BP status in developing CVD. Study participants were categorized into 6 groups according to a combination of TyG index (Normal TyG index, ≤ 8.57; Elevated TyG index, > 8.57) and blood pressure classification (normal, stage 1 hypertension, stage 2 hypertension). We conducted multivariable Cox regression analyses to identify the association of each combination of TyG index and BP status with incident cardiovascular disease: (**A**) cardiovascular disease, (**B**) myocardial infarction, (**C**) stroke. We adjusted the hazard ratio of each combination for Multivariable model adjusted for age, sex, income, physical exercise, educational level, drinking, smoking, diabetes, anti-hypertensive medication, anti-diabetic medication, anti-hyperlipidemic medication, BMI, RHR, LDL-C, UA, Hs-CRP

After a mean follow-up of 13.66 ± 3.24 years, incident CVD, MI, and stroke occurred in 8,205, 1,728, and 6,705, respectively. The participants were stratified into six cohorts according to their TyG index and BP status. Kaplan-Meier curve analysis revealed a significant increase in the prevalence of CVD among various TyG index and BP status groups (*P* < 0.001). Meanwhile, after adjusting for confounding factors, the multivariate Cox proportional hazard model showed that the risk in the elevated TyG index and stage 2 HTN groups was increased 2.58-fold compared with that in the low TyG index and normal/elevated BP groups. In addition, the HR for CVD was 1.41 (95% CI 1.25–1.59) in elevated TyG index group alone, 1.38 (95% CI 1.24–1.54) in stage 1 HTN alone, and 2.35 (95% CI 2.13–2.60) in stage 2 HTN alone. Similar results were obtained in all subgroup analyses (Fig. 1). Multiplicative interactions were statistically significant for CVD (*P* < 0.001). Following adjustments for other variables, three measurements of additive interaction were statistically significant, with RERI at 0.55 (95% CI 0.49–0.62), AP at 32.45% (95% CI 29.88%-35.01%), and SI at 4.61 (95% CI 3.41–6.24).

### Association between BP status and incident CVD based on the TyG index

The relationship between BP status and incident CVD based on the TyG index is summarized in Table 2. The rates of CVD, MI, and stroke increased with an increase in the TyG index. Conversely, the HR for individuals with stage 1 or stage 2 HTN developing CVD decreased with an increase in the TyG index. Additionally, the RRR for individuals with stage 1 or stage 2 HTN developing CVD, MI, and stroke also decreased with an increase in the TyG index. For instance, the RRR for individuals with stage 2 HTN developing CVD was 54.75% in the low TyG index group and 47.09% in the elevated TyG index group.

**Table 2:** Association of BP status with incident CVD according to TyG index

	BP status	Number	Events	Incidence, %	Multivariable model	RRR
CVD						
Normal						
	Normal	15,420	547	3.62	Ref.	Ref.
	Stage 1	14,745	867	6.23	1.36(1.22–1.51)	26.47%
	Stage 2	14,128	1845	14.26	2.21(1.99–2.46)	54.75%
Elevated						
	Normal	8576	528	6.39	Ref.	Ref.
	Stage 1	13,804	1123	8.50	1.23(1.10–1.36)	18.70%
	Stage 2	21,711	3295	16.34	1.89(1.71–2.08)	47.09%
MI						
Normal						
	Normal	15,420	101	0.69	Ref.	Ref.
	Stage 1	14,745	159	1.14	1.30(1.01–1.67)	23.08%
	Stage 2	14,128	329	2.56	1.88(1.47–2.41)	46.81%
Elevated						
	Normal	8576	130	1.57	Ref.	Ref.
	Stage 1	13,804	267	2.05	1.18(0.95–1.45)	15.25%
	Stage 2	21,711	742	3.73	1.56(1.28–1.90)	35.90%
Stroke						
Normal						
	Normal	15,420	457	3.02	Ref.	Ref.
	Stage 1	14,745	724	5.23	1.36(1.20–1.53)	26.47%
	Stage 2	14,128	1566	12.17	2.28(2.03–2.55)	56.14%
Elevated						
	Normal	8576	403	4.92	Ref.	Ref.
	Stage 1	13,804	886	6.71	1.26(1.12–1.42)	20.63%
	Stage 2	21,711	2669	13.31	2.01(1.80–2.24)	50.25%

Multivariable model adjusted for age, sex, income, physical exercise, educational level, drinking, smoking, diabetes, anti-hypertensive medication, anti-diabetic medication, anti-hyperlipidemic medication, BMI, RHR, LDL-C, UA, Hs-CRP. RRR relative risk reduction

### Association between TyG index and incident CVD based on the BP status

The relationship between the TyG index and incident CVD based on BP status is summarized in Table 3. The rates of CVD, MI, and stroke increased with an increase in BP status. Conversely, the HR for individuals at CVD risk decreased with an increase in BP status in the elevated TyG index group. Additionally, the RRR for individuals with CVD, MI, and stroke also decreased with an increase in BP status in the elevated TyG index group (Table 3). For instance, the RRR for individuals with CVD in the elevated TyG index group was 23.08% in individuals with normal/elevated BP, 18.70% in those with stage 1 HTN, and 9.09% in those with stage 2 HTN.

**Table 3:** Association of TyG index with incident CVD according to BP status

	TyG index	Number	Events	Incidence	Multivariable model	RRR	
CVD							
Normal							
	Normal	15,420	547	3.62	Ref.	Ref.	
	Elevated	8576	528	6.39	1.30(1.14–1.48)	23.08%	
Stage 1							
	Normal	14,745	867	6.23	Ref.	Ref.	
	Elevated	13,804	1123	8.50	1.23(1.12–1.36)	18.70%	
Stage 2							
	Normal	14,128	1845	14.26	Ref.	Ref.	
	Elevated	21,711	3295	16.34	1.10(1.03–1.17)	9.09%	
MI							
Normal							
	Normal	15,420	101	0.69	Ref.	Ref.	
	Elevated	8576	130	1.57	1.74(1.31–2.32)	42.53%	
Stage 1							
	Normal	14,745	159	1.14	Ref.	Ref.	
	Elevated	13,804	267	2.05	1.56(1.26–1.93)	35.90%	
Stage 2							
	Normal	14,128	329	2.56	Ref.	Ref.	
	Elevated	21,711	742	3.73	1.37(1.19–1.57)	27.01%	
Stroke							
Normal							
	Normal	15,420	457	3.02	Ref.	Ref.	
	Elevated	8576	403	4.92	1.16(1.01–1.35)	13.79%	
Stage 1							
	Normal	14,745	724	5.23	Ref.	Ref.	
	Elevated	13,804	886	6.71	1.16(1.04–1.29)	13.79%	
Stage 2							
	Normal	14,128	1566	12.17	Ref.	Ref.	
	Elevated	21,711	2669	13.31	1.04(0.97–1.12)	3.85%	

Multivariable model adjusted for age, sex, income, physical exercise, educational level, drinking, smoking, diabetes, anti-hypertensive medication, anti-diabetic medication, anti-hyperlipidemic medication, BMI, RHR, LDL-C, UA, Hs-CRP. RRR relative risk reduction

### Sensitivity analyses

Five sensitivity analyses were performed to validate these findings (Table S3-S6, Figure S2-S5). It was found that the relationships with incident CVD risk remained consistent after excluding participants with CVD events within the initial two years of follow-up, those receiving lipid/glucose-lowering medication, and those with diabetes. Similar results were observed in competing risk regression models. Additionally, the interactive effect between BP status and glycemic status, as defined by FBG levels, on CVD risk showed no significant difference.

## Discussion

The current prospective cohort study involving 88,384 participants from the Kailuan study revealed a notable interaction between BP status and the TyG index in relation to the risk of CVD. Additionally, stratification analysis further confirmed that the relative contribution of HTN to CVD development decreased with a decrease TyG index. In addition, the TyG index declined with increasing BP status. Our study provides a nuanced understanding of the interplay between these factors and their impact on CVD risk assessment, aiding in the targeted identification of high-risk individuals and providing significant implications for both clinical practice and public health strategies.

Furthermore, our data revealed a novel and significant combined effect of BP status and TyG index on the risk of CVD, demonstrating that the incidence risk of CVD is substantially higher in individuals with this combined effect than in those with elevated TyG index or HTN alone. Numerous studies have demonstrated that BP status and TyG index are independent risk factors for CVD in the general population [9, 10]. Theoretically, it might be hypothesized that a combined effect of the two risk factors may contribute to the development of CVD. However, this conjecture remains to be confirmed. Our findings confirmed that participants with HTN (stage 1 and stage 2) combined with elevated TyG index were at a higher risk of CVD. Our previous study also showed that hypertensive patients with a long-term elevated TyG index were at a heightened risk of stroke [12]. Consistent with our results, Wang et al. demonstrated that individuals with a higher TyG index exhibited increased arterial stiffness, and the TyG index was positively and independently associated with arterial stiffness in patients with stage 1 HTN [17]. The present study revealed that elevated TyG index had a stronger association with the occurrence of MI in individuals with variations in both BP status and TyG index, while HTN was more closely linked to the risk of stroke. This phenomenon may be attributed to various pathogenic mechanisms. The rapid improvement of the TyG index may not promptly mitigate IR, a crucial factor in the progression of metabolic dysregulation, affecting glucose levels, lipid metabolism, and vascular endothelial function. Elevated BP is more prone to inducing vascular impairments, resulting in cerebrovascular dysfunction and subsequent stroke.

A survey of HTN, hyperglycemia, and dyslipidemia prevalence in China indicated that the prevalence of HTN in middle-aged and elderly people with diabetes was 12.3%, and the prevalence of comorbidity increased gradually with age [18]. A retrospective observation cohort study from Japan reported a significant interaction between HTN and diabetes [19]. Similar results were observed in our sensitivity analysis, further confirming the reliability of our results. IR is a crucial pathophysiologic foundation and serves as the “soil” in the pathogenetic process of diabetes, frequently occurring in prediabetic patients. TyG index is a surrogate marker of IR deemed to provide better predictive performance in CVD. The current study utilized the TyG index to evaluate IR to explore the interaction between IR and HTN on the risk of CVD. It was found that the HR for CVD increased 1.41-fold from the low group to the elevated group in normotensive participants, the increased CVD risk was more prominent in stages 1 and 2 patients. Elevated TyG index was associated with increased CVD risk, regardless of whether the BP levels were within the normal range. This result suggests that in addition to IR improvement, individualized antihypertensive therapy is also necessary.

Interaction models suggested the presence of positive additive interaction and multiplicative interaction between HTN and elevated TyG index on CVD risk. That is, the interaction of the two factors was higher than the influence of each single factor. A few other studies have also reached similar conclusions. A population-based study from the National Health and Nutrition Examination Survey found that the combination of low TyG index and low SBP (< 120 and < 130 mmHg) further reduced all-cause and cardiovascular mortality risk than the other groups, with a more significant effect on cardiovascular mortality [20]. Several potential mechanisms may explain the concomitant relationship between the TyG index and BP status in the development of CVD. Chronic HTN has been shown to induce target organ damage, such as cardiac, cerebral, and renal complications, thereby elevating the risk of CVD. Conversely, elevated levels of the TyG index are indicative of IR, a factor implicated in the pathogenesis of CVD [21]. IR can increase blood glucose levels, resulting in inflammation, oxidative stress, and dyslipidemia, all of which can contribute to the progression of CVD [22]. Additionally, IR can disrupt the normal secretion of nitric oxide and impair endothelial function [23, 24]. IR and HTN can expedite the development of cardiovascular damage, thereby heightening the likelihood of CVD. Understanding the potential synergistic, additive, or antagonistic impact of elevated TyG index and HTN on CVD risk is imperative. This knowledge may greatly impact the assessment of CVD risk by clinicians and the development of more personalized and effective prevention strategies.

Moreover, hierarchical stratified analysis was performed by BP levels and the TyG index. The risk of developing CVD varied depending on the level of exposure to the TyG index in different BP groups. Similarly, within different levels of the TyG index, the risk of developing CVD varied according to different BP groups. For example, in the low TyG index group, the risk of CVD was higher in patients with stage 2 HTN than in those with elevated TyG index. RRR was calculated to further explain this interesting phenomenon. As anticipated, the RRR was higher in patients with stage 2 than in those with elevated TyG index. That is, the potential benefit of HTN treatment would be greater in individuals with a low TyG index than in those with an elevated TyG index. This discovery has important practical implications for clinical practice and risk stratification. Utilizing the TyG index and BP status to identify individuals at a heightened risk enables the implementation of tailored preventive measures. For those at increased risk, lifestyle changes, including weight management, dietary modifications, and enhanced physical activity, are strongly recommended. Moreover, healthcare providers should contemplate implementing more assertive strategies for managing cardiovascular risk factors, including HTN and abnormalities in glucose and lipid metabolism, among individuals in this particular demographic.

The strengths of our study lie in its prospective design, large sample size, and long follow-up period. However, this study has some limitations. First, we checked the IR by calculating the TyG index rather than using the gold standard method, hyperinsulinemic-euglycemic clamp. Previous studies demonstrated that the TyG index is strongly correlated with homeostatic model assessment for insulin resistance (HOMA-IR) and the hyperinsulinemic-euglycemic clamp, even outperforming the HOMA-IR [25, 26]. Second, we did not obtain precise information on glucose-lowering medications for patients. To assess the potential influence of diabetes, we performed a sensitivity analysis by excluding participants taking diabetes medications or diagnosed with diabetes. This exclusion did not alter our main findings. Third, more evidence suggests that the TyG index is not a permanent state as it is dynamic. However, our analysis was based solely on baseline data. Fourth, while our multivariate analysis adjusted for known confounders, the possibility of residual confounding by unmeasured variables remains a limitation. Finally, the lack of data on the cause of death hampered further analysis of the association between the interaction of TyG index and BP status and the risk of cardiovascular death.

## Conclusion

Our study demonstrated that a significant interaction between TyG index and BP status on the risk of CVD. Enhancing IR and implementing aggressive BP management strategies may serve as effective preventive measures for CVD and mitigate associated risks.

### Electronic supplementary material

Below is the link to the electronic supplementary material.

Supplementary Material 1

## Data Availability

No datasets were generated or analysed during the current study.

## References

[1] Leong PJD, McKee M, et al. Reducing the Global Burden of Cardiovascular Disease, Part 1: the epidemiology and risk factors. Circul Res. 2017;121(6):677–94. 10.1161/CIRCRESAHA.117.311849 28860318

[2] Gregory A, Roth GA, Mensah, Catherine O, Johnson et al. Global Burden of Cardiovascular diseases and Risk factors, 1990–2019: Update from the GBD 2019 study. J Am Coll Cardiol 2020 12 22;76(25):2982–3021. 10.1016/j.jacc.2020.11.010 PMC7755038 33309175

[3] Global Cardiovascular Risk Consortium, Magnussen C, Francisco M, Ojeda et al. Global Effect of Modifiable Risk Factors on Cardiovascular Disease and Mortality. The New England journal of medicine 2023 Aug 26.

[4] Xin D, Patel A, Craig S, Anderson et al. Epidemiology of Cardiovascular Disease in China and opportunities for Improvement: JACC International. J Am Coll Cardiol 2019 06 25;73(24):3135–47. 10.1016/j.jacc.2019.04.036 31221263

[5] Dong Zhao J, Liu M, Wang, et al. Epidemiology of cardiovascular disease in China: current features and implications. Nat Rev Cardiol. 2019;04(4):203–12. 10.1038/s41569-018-0119-4 30467329

[6] Odayme Quesada B, Claggett F, Rodriguez et al. Associations of Insulin Resistance With Systolic and Diastolic Blood Pressure: A Study From the HCHS/SOL. Hypertension (Dallas, Tex.: 1979) 2021 09;78(3):716–725. 10.1161/HYPERTENSIONAHA.120.16905 PMC8650976 34379440

[7] Bizhong Che C, Zhong R, Zhang, et al. Triglyceride-glucose index and triglyceride to high-density lipoprotein cholesterol ratio as potential cardiovascular disease risk factors: an analysis of UK biobank data. Cardiovasc Diabetol. 2023;02(16):34. 10.1186/s12933-023-01762-2 PMC9936712 36797706

[8] Sangmo Hong K, Han C-Y, Park. The triglyceride glucose index is a simple and low-cost marker associated with atherosclerotic cardiovascular disease: a population-based study. BMC medicine 2020 11 25;18(1):361. 10.1186/s12916-020-01824-2 PMC7687762 33234146

[9] Niloofar Barzegar M, Tohidi M, Hasheminia, et al. The impact of triglyceride-glucose index on incident cardiovascular events during 16 years of follow-up: Tehran lipid and glucose study. Cardiovasc Diabetol. 2020;09(29):155. 10.1186/s12933-020-01121-5 PMC7526412 32993633

[10] Haozhe Cui Q, Liu Y, Wu, et al. Cumulative triglyceride-glucose index is a risk for CVD: a prospective cohort study. Cardiovasc Diabetol. 2022;02(10):22. 10.1186/s12933-022-01456-1 PMC8830002 35144621

[11] Nikolaus Marx M, Federici K, Schütt et al. 2023 ESC Guidelines for the management of cardiovascular disease in patients with diabetes. European heart journal 2023;44(39):4043–4140. 10.1093/eurheartj/ehad192 37622663

[12] Zegui Huang X, Ding Q, Yue, et al. Triglyceride-glucose index trajectory and stroke incidence in patients with hypertension: a prospective cohort study. Cardiovasc Diabetol. 2022;07(27):141. 10.1186/s12933-022-01577-7 PMC9331781 35897017

[13] Qian Liu H, Cui S, Chen, et al. Association of baseline life’s essential 8 score and trajectories with carotid intima-media thickness. Front Endocrinol (Lausanne). 2023;14:1186880. 37334294 10.3389/fendo.2023.1186880 PMC10272710

[14] Whelton PK, Carey RM, Aronow WS, etal, et al. 2017 ACC/AHA/AAPA/ABC/ACPM/AGS/APhA/ASH/ASPC/NMA/PCNA Guideline for the Prevention, detection, evaluation, and management of high blood pressure in adults: a report of the American College of Cardiology/American Heart Association Task Force on Clinical Practice guidelines [published correction appears in J am Coll Cardiol. 2018;71(19):2275–2279]. J Am Coll Cardiol. 2018;71(19):e127–248. 29146535 10.1016/j.jacc.2017.11.006

[15] Andersson T, Alfredsson L, Källberg H, Zdravkovic S, Ahlbom A. Calculating measures of biological interaction. Eur J Epidemiol. 2005;20(7):575–9. 16119429 10.1007/s10654-005-7835-x

[16] Knol MJ, VanderWeele TJ, Groenwold RH, Klungel OH, Rovers MM, Grobbee DE. Estimating measures of interaction on an additive scale for preventive exposures. Eur J Epidemiol. 2011;26(6):433–8. 21344323 10.1007/s10654-011-9554-9 PMC3115067

[17] Wu Z, Zhou D, Liu Y, et al. Association of TyG index and TG/HDL-C ratio with arterial stiffness progression in a non-normotensive population. Cardiovasc Diabetol. 2021;20(1):134. 34229681 10.1186/s12933-021-01330-6 PMC8262008

[18] Yu N, Zhang M, Zhang X, et al. Study on the status and influencing factors of comorbidity of hypertension, diabetes, and dyslipidemia among middle-aged and elderly Chinese adults. Zhong Hua Liu Xing Bing Xue Za Zhi. 2023;44(02):196–204.

[19] Suzuki Y, Kaneko H, Yano Y, et al. Interaction of blood pressure and glycemic status in developing Cardiovascular Disease: analysis of a Nationwide Real-World database. J Am Heart Assoc. 2023;12(1):e026192. 36565182 10.1161/JAHA.122.026192 PMC9973580

[20] Yu Y, Gu M, Huang H, et al. Combined association of triglyceride-glucose index and systolic blood pressure with all-cause and cardiovascular mortality among the general population. J Transl Med. 2022;20(1):478. 36266665 10.1186/s12967-022-03678-z PMC9583494

[21] Khan SH, Sobia F, Niazi NK, Manzoor SM, Fazal N, Ahmad F. Metabolic clustering of risk factors: evaluation of triglyceride-glucose index (TyG index) for evaluation of insulin resistance. Diabetol Metab Syndr. 2018;10:74. 30323862 10.1186/s13098-018-0376-8 PMC6173832

[22] Yang Q, Vijayakumar A, Kahn BB. Metabolites as regulators of insulin sensitivity and metabolism. Nat Rev Mol Cell Biol. 2018;19(10):654–72. 30104701 10.1038/s41580-018-0044-8 PMC6380503

[23] Molina MN, Ferder L, Manucha W. Emerging role of nitric oxide and heat shock proteins in insulin resistance. Curr Hypertens Rep. 2016;18(1):1. 26694820 10.1007/s11906-015-0615-4

[24] Nishikawa T, Kukidome D, Sonoda K et al. Impact of mitochondrial ROS production in the pathogenesis of insulin resistance. Diabetes Res Clin Pract. 2007;77. 10.1016/j.diabres.2007.01.071 17481767

[25] Guerrero-Romero F, Simental-Mendía LE, González-Ortiz M, et al. The product of triglycerides and glucose, a simple measure of insulin sensitivity. Comparison with the euglycemic-hyperinsulinemic clamp. J Clin Endocrinol Metab. 2010;95(7):3347–51. 20484475 10.1210/jc.2010-0288

[26] Vasques AC, Novaes FS, de Oliveira Mda S, et al. TyG index performs better than HOMA in a Brazilian population: a hyperglycemic clamp validated study. Diabetes Res Clin Pract. 2011;93(3):e98–100. 21665314 10.1016/j.diabres.2011.05.030

