# Supplementary material for: Interacting and joint effects of triglyceride-glucose index and blood pressure on cardiovascular diseases risk: a prospective cohort study
Source: Diabetol Metab Syndr. 2024 Aug 2;16:188. doi: 10.1186/s13098-024-01433-6 (PMC11297715; doi:10.1186/s13098-024-01433-6)
Supplement: Supplementary file 1 — Supplementary Material 1 [file 13098_2024_1433_MOESM1_ESM.docx]

Table S1. Baseline characteristics of participants by TyG index.

|  | TyG index |  | P |
| --- | --- | --- | --- |
|  | Normal(≤8.57) | Elevated(>8.57) |  |
| N | 44293 | 44091 | ＜0.01 |
| Age, years | 50.34±13.07 | 51.79±11.63 | ＜0.01 |
| Male, % | 76.05 | 82.69 | ＜0.01 |
| SBP, mmHg | 126.33±20.25 | 133.93±21.01 | ＜0.01 |
| BMI, kg/m2 | 23.99±3.32 | 26.02±3.38 | ＜0.01 |
| RHR, bpm/min | 72.87±9.82 | 74.80±10.34 | ＜0.01 |
| LDL-C, mmol | 2.28(1.76-2.89) | 2.40(1.92-2.89) | ＜0.01 |
| UA, U/L | 275.27±75.60 | 303.02±87.50 | ＜0.01 |
| Hs-CRP, mg/dl | 0.66(0.25-1.80) | 0.92(0.37-2.30) | ＜0.01 |
| TG, mmol | 0.89±0.27 | 2.44±1.59 | ＜0.01 |
| FBG, mmol | 4.95±0.73 | 5.98±2.10 | ＜0.01 |
| TyG index | 8.11±0.34 | 9.18±0.52 | ＜0.01 |
| Smoking, % | 29.38 | 32.22 | ＜0.01 |
| Drinking, % | 16.57 | 19.20 | ＜0.01 |
| Diabetes, % | 1.74 | 16.02 | ＜0.01 |
| Active exercise, % | 15.21 | 15.11 | ＜0.01 |
| BP medicine, % | 7.39 | 13.63 | ＜0.01 |
| Lipid medicine, % | 0.45 | 1.10 | ＜0.01 |
| Glucose medicine, % | 0.72 | 3.70 | ＜0.01 |
| High school or above, % | 22.24 | 18.75 | ＜0.01 |

SBP systolic blood pressure, BMI body mass index, RHR resting heart rate, LDL low-density lipoprotein, UA uric acid, Hs-CRP high-sensitivity C-reactive protein, TG triglyceride, FBG fasting blood glucose, TyG triglyceride glucose.

Table S2. Baseline characteristics of participants by BP status.

|  | BP status | | | P |
| --- | --- | --- | --- | --- |
|  | Normal BP | Stage 1 | Stage 2 |  |
| N | 23996 | 28549 | 35839 | ＜0.01 |
| Age, years | 46.47±12.75 | 49.41±11.74 | 55.46±11.17 | ＜0.01 |
| Male, % | 68.85 | 80.65 | 85.37 | ＜0.01 |
| SBP, mmHg | 110.33±10.04 | 123.15±8.10 | 148.93±17.63 | ＜0.01 |
| BMI, kg/m2 | 23.72±3.24 | 24.78±3.31 | 26.03±3.49 | ＜0.01 |
| RHR, bpm/min | 72.06±9.66 | 73.48±9.63 | 75.29±10.59 | ＜0.01 |
| LDL-C, mmol | 2.23(1.76-2.72) | 2.32(1.83-2.81) | 2.40(1.90-2.91) | ＜0.01 |
| UA, U/L | 279.88±78.84 | 284.07±79.29 | 299.32±87.22 | ＜0.01 |
| Hs-CRP, mg/dl | 0.69(0.27-1.80) | 0.70(0.26-1.80) | 0.99(0.38-2.50) | ＜0.01 |
| TG, mmol | 1.35±1.09 | 1.65±1.39 | 1.89±1.49 | ＜0.01 |
| FBG, mmol | 5.21±1.42 | 5.38±1.52 | 5.70±1.85 | ＜0.01 |
| TyG index | 8.42±0.64 | 8.63±0.68 | 8.82±0.69 | ＜0.01 |
| Smoking, % | 32.26 | 31.14 | 29.55 | ＜0.01 |
| Drinking, % | 14.83 | 17.79 | 20.00 | ＜0.01 |
| Diabetes, % | 4.94 | 6.80 | 13.14 | ＜0.01 |
| Active exercise, % | 13.39 | 13.01 | 18.05 | ＜0.01 |
| BP medicine, % | 0 | 0 | 25.90 | ＜0.01 |
| Lipid medicine, % | 0.46 | 0.26 | 1.40 | ＜0.01 |
| Glucose medicine, % | 1.50 | 1.33 | 3.38 | ＜0.01 |
| High school or above, % | 32.78 | 18.84 | 13.60 | ＜0.01 |

SBP systolic blood pressure, BMI body mass index, RHR resting heart rate, LDL low-density lipoprotein, UA uric acid, Hs-CRP high-sensitivity C-reactive protein, TG triglyceride, FBG fasting blood glucose, TyG triglyceride glucose.

Table S3. Association of BP status with incident CVD according to TyG index-sensitivity analysis.

|  | BP status | Analysis 1 | Analysis 2 | Analysis 3 | Analysis 4 |
| --- | --- | --- | --- | --- | --- |
| CVD |  |  |  |  |  |
| Normal |  |  |  |  |  |
|  | Normal | Ref. | Ref. | Ref. | Ref. |
|  | Stage 1 | 1.38(1.23-1.55) | 1.35(1.21-1.50) | 1.36(1.22-1.52) | 1.36(1.22-1.51) |
|  | Stage 2 | 2.19(1.96-2.45) | 2.21(1.98-2.45) | 2.22(2.00-2.48) | 2.21(1.99-2.46) |
| Elevated |  |  |  |  |  |
|  | Normal | Ref. | Ref. | Ref. |  |
|  | Stage 1 | 1.24(1.12-1.39) | 1.23(1.10-1.37) | 1.28(1.14-1.45) | 1.23(1.10-1.36) |
|  | Stage 2 | 1.87(1.70-2.07) | 1.88(1.70-2.08) | 1.99(1.78-2.23) | 1.89(1.71-2.08) |
| MI | | | | |  |
| Normal |  |  |  |  |  |
|  | Normal | Ref. | Ref. | Ref. | Ref. |
|  | Stage 1 | 1.38(1.05-1.80) | 1.30(1.01-1.68) | 1.31(1.02-1.70) | 1.30(1.01-1.67) |
|  | Stage 2 | 1.92(1.47-2.51) | 1.85(1.45-2.38) | 1.91(1.48-2.45) | 1.88(1.47-2.40) |
| Elevated |  |  |  |  |  |
|  | Normal | Ref. | Ref. | Ref. |  |
|  | Stage 1 | 1.24(0.99-1.54) | 1.16(0.93-1.44) | 1.22(0.96-1.55) | 1.18(0.95-1.45) |
|  | Stage 2 | 1.61(1.30-1.99) | 1.52(1.23-1.86) | 1.60(1.28-2.02) | 1.56(1.27-1.91) |
| Stroke | | | | |  |
| Normal |  |  |  |  |  |
|  | Normal | Ref. | Ref. | Ref. | Ref. |
|  | Stage 1 | 1.37(1.21-1.55) | 1.34(1.19-1.51) | 1.36(1.20-1.53) | 1.36(1.20-1.53) |
|  | Stage 2 | 2.24(1.99-2.53) | 2.27(2.02-2.55) | 2.29(2.04-2.57) | 2.28(2.03-2.56) |
| Elevated |  |  |  |  |  |
|  | Normal | Ref. | Ref. | Ref. |  |
|  | Stage 1 | 1.27(1.12-1.43) | 1.27(1.13-1.44) | 1.33(1.17-1.53) | 1.26(1.12-1.42) |
|  | Stage 2 | 1.97(1.75-2.21) | 2.01(1.80-2.26) | 2.15(1.89-2.44) | 2.01(1.79-2.24) |

Note1: Multivariable model adjusted for age, sex, income, physical exercise, educational level, drinking, smoking, diabetes, lipid medicine, glucose medicine, BP medicine, BMI, RHR, LDL-C, UA, Hs-CRP.

Note2: Analysis1 excluded participants events occurring in the first 2 years of follow-up(n=1327); Analysis2 excluded received treatment with lipid lowering medication or glucose lowering medication at baseline and follow-up(n=2500); Analysis3 excluded participants with diabetes at baseline (n=7834); Analysis4 was competing risk analysis.

Table S4. Association of TyG index with incident CVD according to BP status-sensitivity analysis.

|  | TyG index | Analysis 1 | Analysis 2 | Analysis 3 | Analysis 4 |
| --- | --- | --- | --- | --- | --- |
| CVD |  |  |  |  |  |
| Normal BP |  |  |  |  |  |
|  | Normal | Ref. | Ref. | Ref. | Ref. |
|  | Elevated | 1.34(1.16-1.53) | 1.29(1.13-1.47) | 1.28(1.17-1.47) | 1.30(1.14-1.48) |
| Stage 1 |  |  |  |  |  |
|  | Normal | Ref. | Ref. | Ref. | Ref. |
|  | Elevated | 1.25(1.13-1.38) | 1.25(1.14-1.38) | 1.23(1.12-1.36) | 1.23(1.12-1.36) |
| Stage 2 |  |  |  |  |  |
|  | Normal | Ref. | Ref. | Ref. | Ref. |
|  | Elevated | 1.12(1.04-1.19) | 1.10(1.03-1.17) | 1.08(1.02-1.16) | 1.10(1.03-1.17) |
| MI | | | | |  |
| Normal BP |  |  |  |  |  |
|  | Normal | Ref. | Ref. | Ref. | Ref. |
|  | Elevated | 1.86(1.37-2.52) | 1.73(1.30-2.32) | 1.75(1.30-2.35) | 1.74(1.31-2.31) |
| Stage 1 |  |  |  |  |  |
|  | Normal | Ref. | Ref. | Ref. | Ref. |
|  | Elevated | 1.63(1.31-2.04) | 1.55(1.25-1.92) | 1.57(1.27-1.95) | 1.56(1.26-1.93) |
| Stage 2 |  |  |  |  |  |
|  | Normal | Ref. | Ref. | Ref. | Ref. |
|  | Elevated | 1.44(1.23-1.67) | 1.38(1.19-1.59) | 1.35(1.17-1.57) | 1.37(1.19-1.57) |
| Stroke | | | | |  |
| Normal BP |  |  |  |  |  |
|  | Normal | Ref. | Ref. | Ref. | Ref. |
|  | Elevated | 1.20(1.03-1.39) | 1.15(0.99-1.34) | 1.15(0.98-1.34) | 1.16(1.01-1.35) |
| Stage 1 |  |  |  |  |  |
|  | Normal | Ref. | Ref. | Ref. | Ref. |
|  | Elevated | 1.16(1.04-1.29) | 1.17(1.05-1.31) | 1.15(1.03-1.29) | 1.16(1.04-1.29) |
| Stage 2 |  |  |  |  |  |
|  | Normal | Ref. | Ref. | Ref. | Ref. |
|  | Elevated | 1.05(0.98-1.13) | 1.04(0.97-1.12) | 1.03(0.96-1.10) | 1.04(0.97-1.12) |

Note1: Multivariable model adjusted for age, sex, income, physical exercise, educational level, drinking, smoking, diabetes, lipid medicine, glucose medicine, BP medicine, BMI, RHR, LDL-C, UA, Hs-CRP.

Note2: Analysis1 excluded participants events occurring in the first 2 years of follow-up(n=1327); Analysis2 excluded received treatment with lipid lowering medication or glucose lowering medication at baseline and follow-up(n=2500); Analysis3 excluded participants with diabetes at baseline (n=7834); Analysis4 was competing risk analysis.

Table S5. Association of BP status with incident CVD according to glucose status.

|  | BP status | Number | Events | Incidence, % | Multivariable model |
| --- | --- | --- | --- | --- | --- |
| CVD |  |  |  |  |  |
| Normal FBG |  |  |  |  |  |
|  | Normal | 21582 | 845 | 3.99 | Ref. |
|  | Stage 1 | 24498 | 1561 | 6.67 | 1.37(1.26-1.49) |
|  | Stage 2 | 27483 | 3607 | 14.15 | 2.18(2.01-2.36) |
| Prediabetes |  |  |  |  |  |
|  | Normal | 1229 | 84 | 7.45 | Ref. |
|  | Stage 1 | 2110 | 178 | 9.17 | 1.15(0.88-1.49) |
|  | Stage 2 | 3648 | 590 | 17.47 | 1.94(1.52-2.46) |
| Diabetes |  |  |  |  |  |
|  | Normal | 1185 | 146 | 13.77 | Ref. |
|  | Stage 1 | 1941 | 251 | 14.25 | 1.06(0.86-1.30) |
|  | Stage 2 | 4708 | 943 | 22.36 | 1.59(1.32-1.91) |
| MI |  |  |  |  |  |
| Normal FBG |  |  |  |  |  |
|  | Normal | 21582 | 173 | 0.83 | Ref. |
|  | Stage 1 | 24498 | 331 | 1.42 | 1.37(1.14-1.65) |
|  | Stage 2 | 27483 | 706 | 2.78 | 1.83(1.53-2.19) |
| Prediabetes |  |  |  |  |  |
|  | Normal | 1229 | 23 | 2.09 | Ref. |
|  | Stage 1 | 2110 | 36 | 1.98 | 0.88(0.52-1.50) |
|  | Stage 2 | 3648 | 141 | 4.40 | 1.67(1.04-2.68) |
| Diabetes |  |  |  |  |  |
|  | Normal | 1185 | 35 | 2.84 | Ref. |
|  | Stage 1 | 1941 | 59 | 3.15 | 1.06(0.70-1.62) |
|  | Stage 2 | 4708 | 224 | 5.47 | 2.43(0.97-2.10) |
| Stroke |  |  |  |  |  |
| Normal FBG |  |  |  |  |  |
|  | Normal | 21582 | 681 | 3.21 | Ref. |
|  | Stage 1 | 24498 | 1258 | 5.38 | 1.38(1.25-1.51) |
|  | Stage 2 | 27483 | 3002 | 11.83 | 2.28(2.08-2.49) |
| Prediabetes |  |  |  |  |  |
|  | Normal | 1229 | 63 | 5.01 | Ref. |
|  | Stage 1 | 2110 | 145 | 15.07 | 1.24(0.92-1.67) |
|  | Stage 2 | 3648 | 468 | 13.95 | 2.03(1.54-2.68) |
| Diabetes |  |  |  |  |  |
|  | Normal | 1185 | 116 | 11.07 | Ref. |
|  | Stage 1 | 1941 | 207 | 11.84 | 1.08(0.86-1.86) |
|  | Stage 2 | 4708 | 765 | 18.32 | 1.61(1.31-1.99) |

Multivariable model adjusted for age, sex, income, physical exercise, educational level, drinking, smoking, lipid medicine, glucose medicine, BP medicine, BMI, RHR, LDL-C, UA, Hs-CRP.

Table S6. Association of glucose status with incident CVD according to BP status.

|  | FBG status | Number | Events | Incidence, % | Multivariable model |
| --- | --- | --- | --- | --- | --- |
| CVD |  |  |  |  |  |
| Normal BP |  |  |  |  |  |
|  | Normal FBG | 21582 | 845 | 3.99 | Ref. |
|  | Prediabetes | 1229 | 84 | 7.45 | 1.47(1.17-1.84) |
|  | Diabetes | 1185 | 146 | 13.77 | 2.37(1.93-2.91) |
| Stage 1 |  |  |  |  |  |
|  | Normal FBG | 24498 | 1561 | 6.67 | Ref. |
|  | Prediabetes | 2110 | 178 | 9.17 | 1.20(1.03-1.40) |
|  | Diabetes | 1941 | 251 | 14.25 | 1.68(1.44-1.95) |
| Stage 2 |  |  |  |  |  |
|  | Normal FBG | 27483 | 3607 | 14.15 | Ref. |
|  | Prediabetes | 3648 | 590 | 17.47 | 1.22(1.12-1.33) |
|  | Diabetes | 4708 | 943 | 22.36 | 1.49(1.37-1.62) |
| MI |  |  |  |  |  |
| Normal BP |  |  |  |  |  |
|  | Normal FBG | 21582 | 173 | 0.83 | Ref. |
|  | Prediabetes | 1229 | 23 | 2.09 | 1.87(1.20-1.90) |
|  | Diabetes | 1185 | 35 | 2.84 | 2.72(1.79-4.15) |
| Stage 1 |  |  |  |  |  |
|  | Normal FBG | 24498 | 331 | 1.42 | Ref. |
|  | Prediabetes | 2110 | 36 | 1.98 | 1.10(0.78-1.55) |
|  | Diabetes | 1941 | 59 | 3.15 | 1.75(1.27-2.41) |
| Stage 2 |  |  |  |  |  |
|  | Normal FBG | 27483 | 706 | 2.78 | Ref. |
|  | Prediabetes | 3648 | 141 | 4.40 | 1.48(1.23-1.77) |
|  | Diabetes | 4708 | 224 | 5.47 | 1.58(1.32-1.90) |
| Stroke |  |  |  |  |  |
| Normal BP |  |  |  |  |  |
|  | Normal FBG | 21582 | 681 | 3.21 | Ref. |
|  | Prediabetes | 1229 | 63 | 5.01 | 1.38(1.06-1.79) |
|  | Diabetes | 1185 | 116 | 11.07 | 2.38(1.85-2.93) |
| Stage 1 |  |  |  |  |  |
|  | Normal FBG | 24498 | 1258 | 5.38 | Ref. |
|  | Prediabetes | 2110 | 145 | 15.07 | 1.22(1.03-1.45) |
|  | Diabetes | 1941 | 207 | 11.84 | 1.72(1.46-2.04) |
| Stage 2 |  |  |  |  |  |
|  | Normal FBG | 27483 | 3002 | 11.83 | Ref. |
|  | Prediabetes | 3648 | 468 | 13.95 | 1.16(1.05-1.28) |
|  | Diabetes | 4708 | 765 | 18.32 | 1.48(1.35-1.63) |

Multivariable model adjusted for age, sex, income, physical exercise, educational level, drinking, smoking, lipid medicine, glucose medicine, BP medicine, BMI, RHR, LDL-C, UA, Hs-CRP.


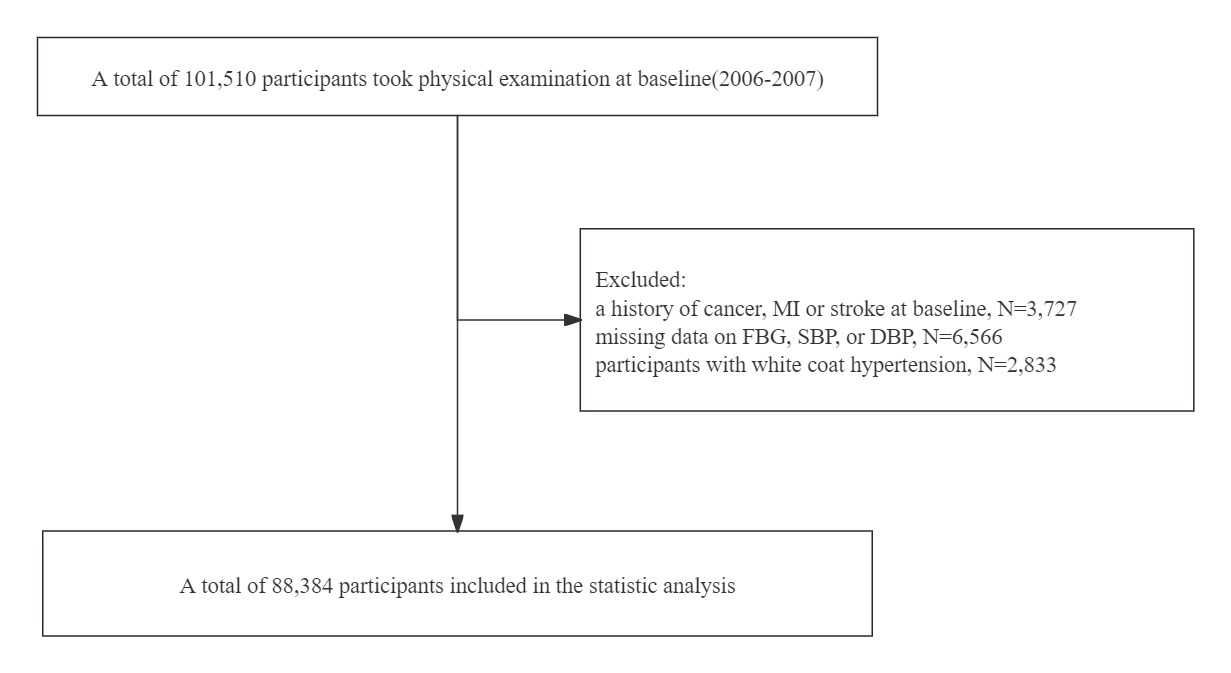


Figure S1. A study flowchart.


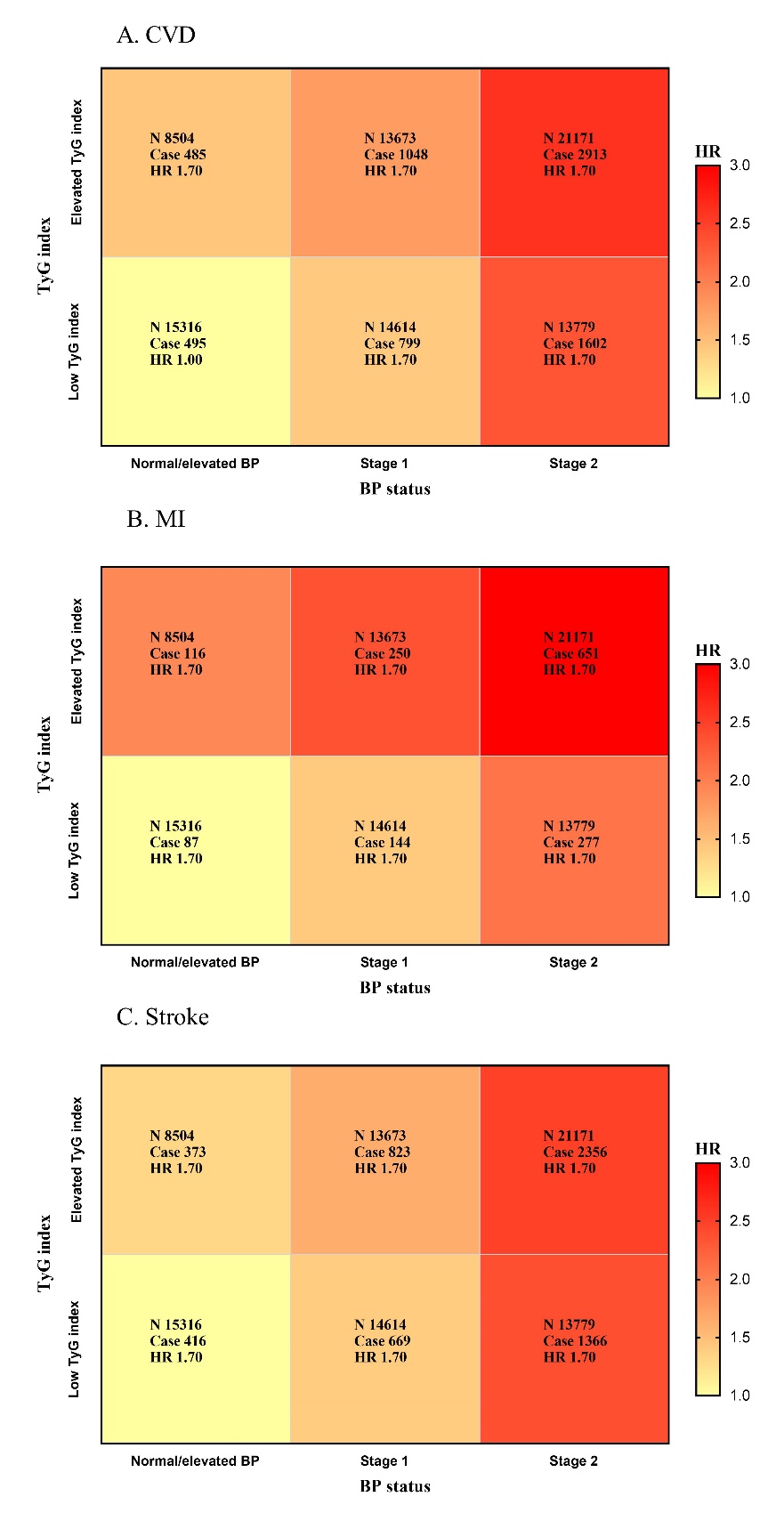


Figure S2. Combination of TyG index and BP status in developing CVD-analysis 1.

Study participants were categorized into 6 groups according to a combination of TyG index (Normal TyG index, ≤8.57; Elevated TyG index, >8.57) and blood pressure classification (normal, stage 1 hypertension, stage 2 hypertension). We conducted multivariable Cox regression analyses to identify the association of each combination of TyG index and BP status with incident cardiovascular disease: (A) cardiovascular disease, (B)myocardial infarction, (C) stroke. We adjusted the hazard ratio of each combination for age, sex, income, physical exercise, educational level, drinking, smoking, lipid medicine, glucose medicine, BP medicine, BMI, RHR, LDL-C, UA, Hs-CRP. Analysis1 excluded participants events occurring in the first 2 years of follow-up(n=1327).


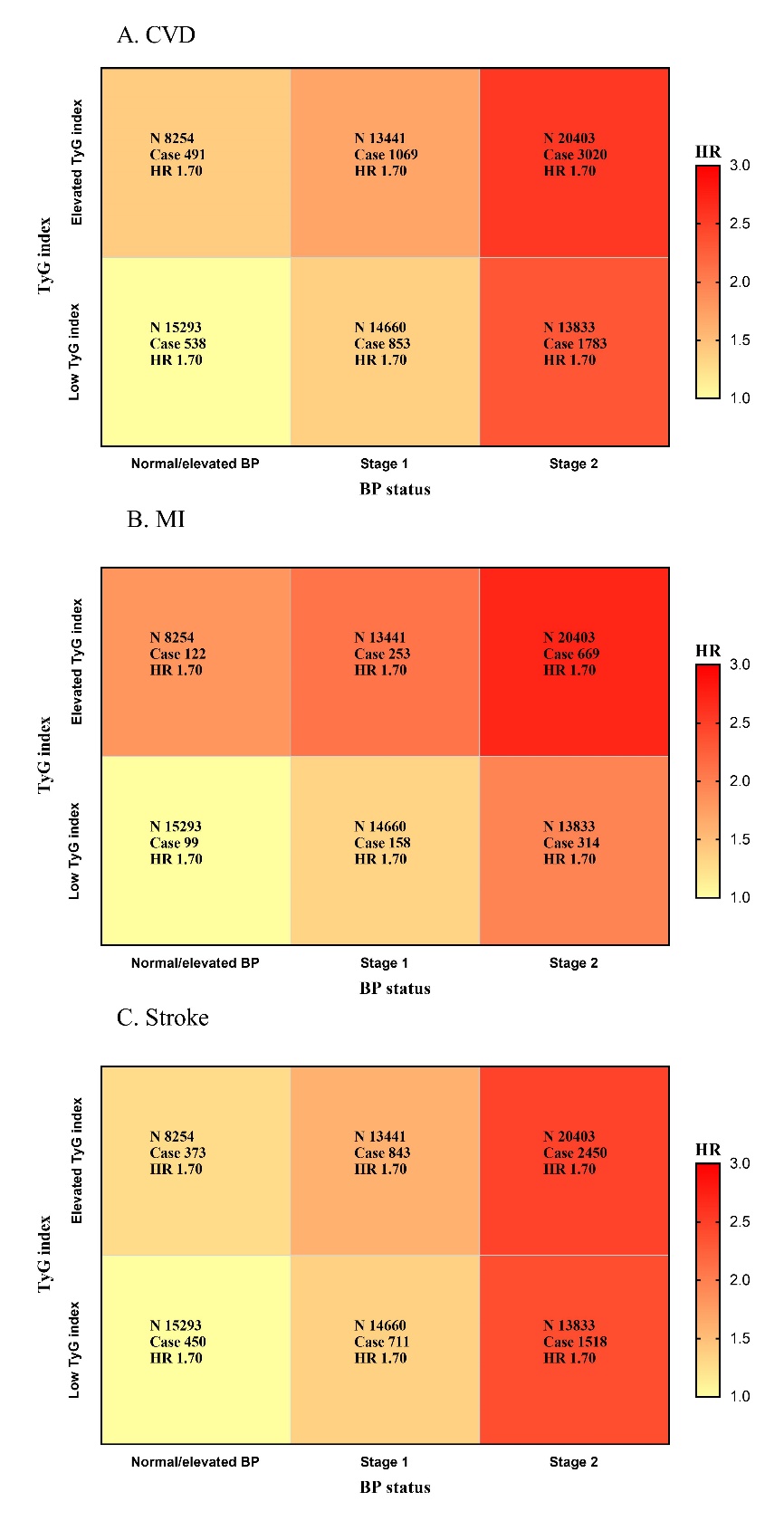


Figure S3. Combination of TyG index and BP status in developing CVD-analysis 2.

Study participants were categorized into 6 groups according to a combination of TyG index (Normal TyG index, ≤8.57; Elevated TyG index, >8.57) and blood pressure classification (normal, stage 1 hypertension, stage 2 hypertension). We conducted multivariable Cox regression analyses to identify the association of each combination of TyG index and BP status with incident cardiovascular disease: (A) cardiovascular disease, (B)myocardial infarction, (C) stroke. We adjusted the hazard ratio of each combination for age, sex, income, physical exercise, educational level, drinking, smoking, lipid medicine, glucose medicine, BP medicine, BMI, RHR, LDL-C, UA, Hs-CRP. Analysis2 excluded received treatment with lipid lowering medication or glucose lowering medication at baseline and follow-up(n=2500).


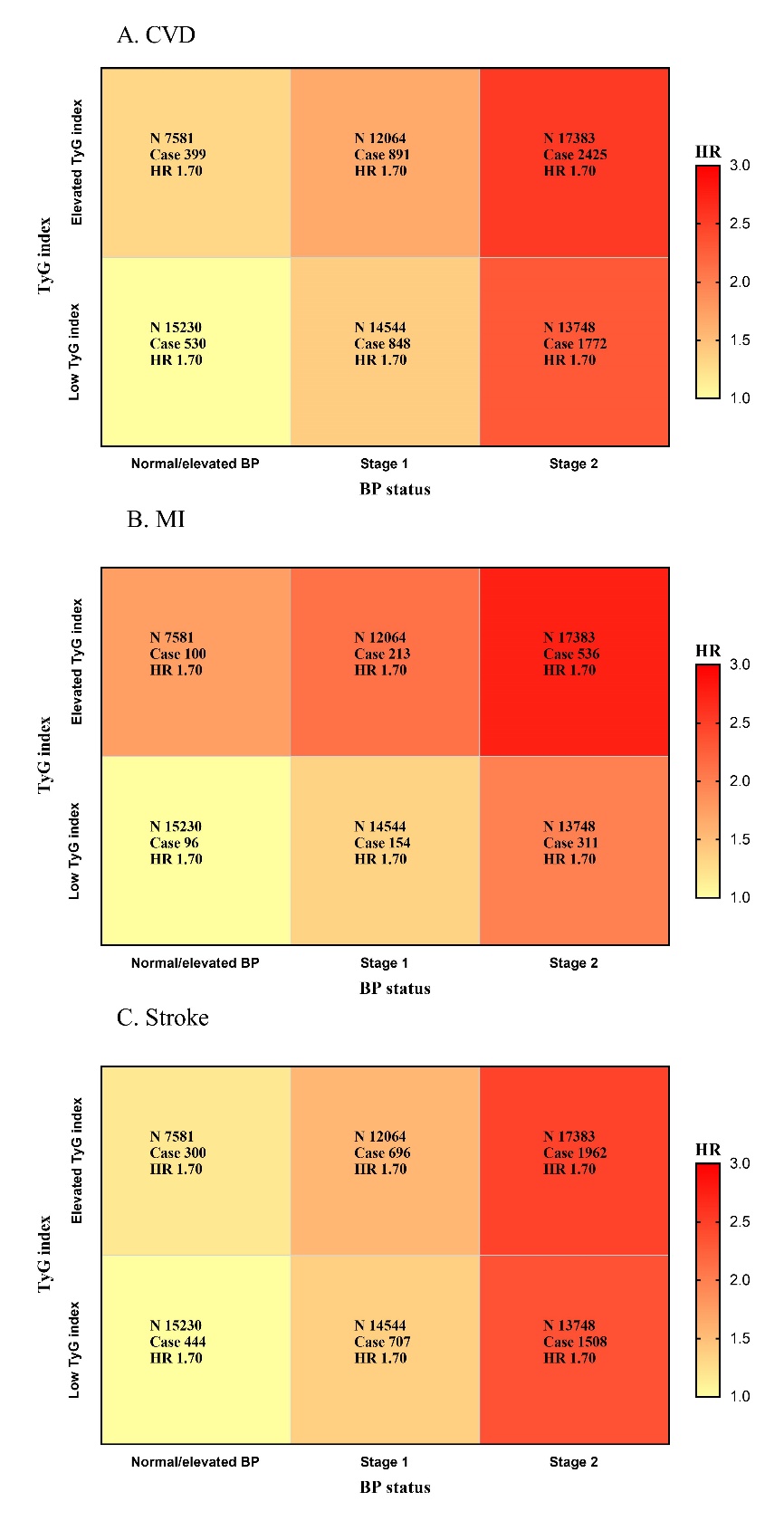


Figure S4. Combination of TyG index and BP status in developing CVD-analysis 3.

Study participants were categorized into 6 groups according to a combination of TyG index (Normal TyG index, ≤8.57; Elevated TyG index, >8.57) and blood pressure classification (normal, stage 1 hypertension, stage 2 hypertension). We conducted multivariable Cox regression analyses to identify the association of each combination of TyG index and BP status with incident cardiovascular disease: (A) cardiovascular disease, (B)myocardial infarction, (C) stroke. We adjusted the hazard ratio of each combination for age, sex, income, physical exercise, educational level, drinking, smoking, lipid medicine, glucose medicine, BP medicine, BMI, RHR, LDL-C, UA, Hs-CRP. Analysis3 excluded participants with diabetes at baseline (n=7834).


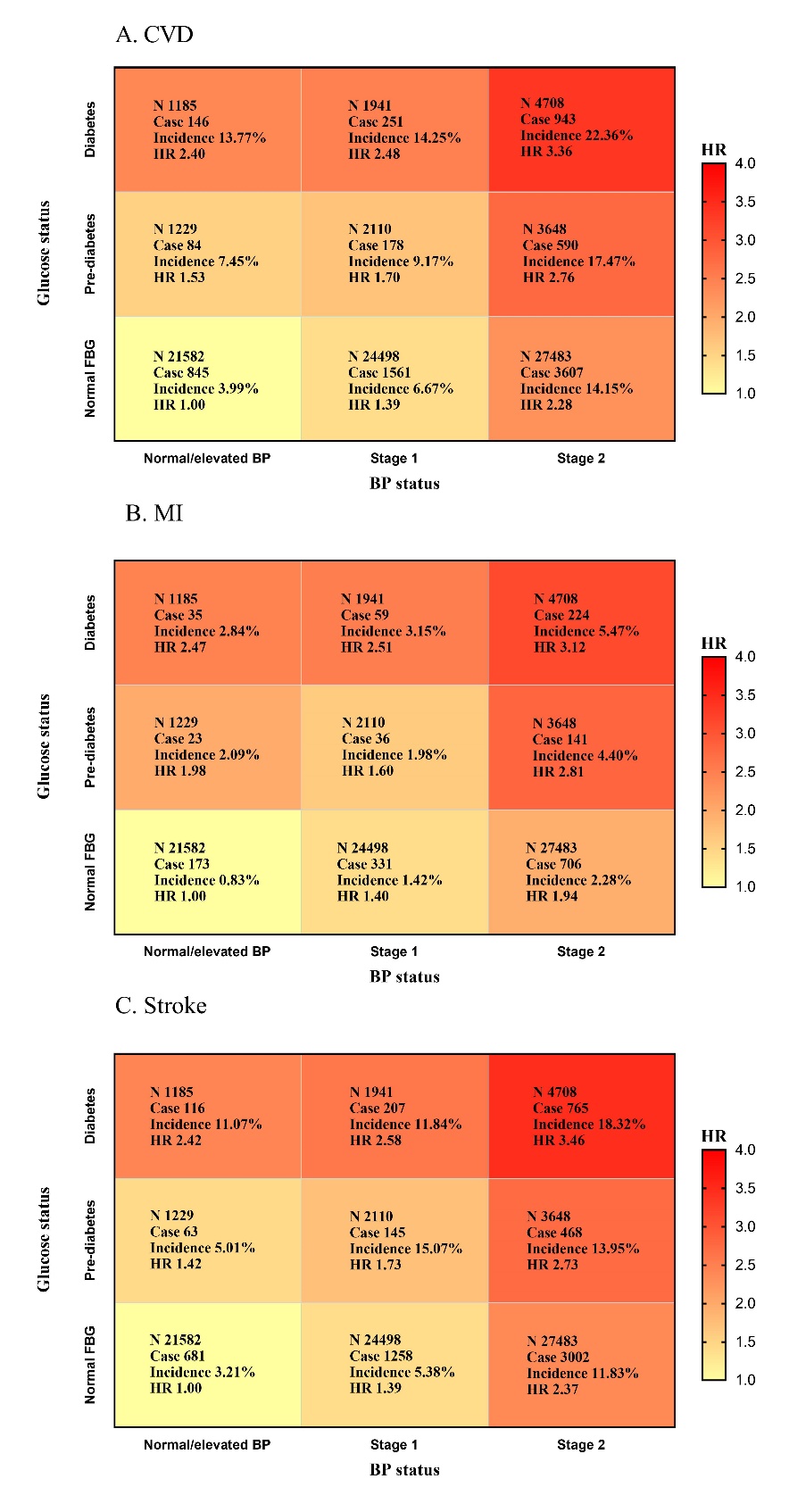


Figure S5. Combination of glucose status and BP status in developing CVD.

Study participants were categorized into 9 groups according to a combination of glucose status(normal, prediabetes and diabetes) and blood pressure classification (normal, stage 1 hypertension, stage 2 hypertension). We conducted multivariable Cox regression analyses to identify the association of each combination of glucose and BP status with incident cardiovascular disease: (A) cardiovascular disease, (B)myocardial infarction, (C) stroke. We adjusted the hazard ratio of each combination for age, sex, income, physical exercise, educational level, drinking, smoking, lipid medicine, glucose medicine, BP medicine, BMI, RHR, LDL-C, UA, Hs-CRP.
